# Abutment Pressure Distribution Law and Support Analysis of Super Large Mining Height Face

**DOI:** 10.3390/ijerph20010227

**Published:** 2022-12-23

**Authors:** Libo Zhang, Wenlong Shen, Xuelong Li, Yabo Wang, Qizhi Qin, Xutao Lu, Tianxi Xue

**Affiliations:** 1 College of Energy and Mining Engineering, Shandong University of Science and Technology, Qingdao 266590, China; 2 State and Local Joint Engineering Laboratory for Gas Drainage & Ground Control of Deep Mines, School of Energy Science and Engineering, Henan Polytechnic University, Jiaozuo 454000, China; 3 Shandong Energy Group Co., Ltd., Jinan 250014, China; 4 Jiaojia Gold Mine, Shandong Gold Mining Industry (Laizhou) Co., Ltd., Laizhou 261441, China

**Keywords:** super large mining height, abutment pressure, rock pressure behavior, surrounding rock control, support design

## Abstract

Under the condition of the shallow coal seam, the laws of roof activity after large mining height longwall face mining and prevention measures for large-area roof weighting are problems that need to be solved urgently. In the background of the super large mining height working face in the upper 108 working face of Jinjitan Coal Mine 12-2, the spatial distribution characteristics of the development and change of the mining-induced abutment pressure and the related support design in the 8.2 m super large mining height and fully mechanized mining face were conducted. It reveals the distribution characteristics of the dynamic stress field and internal and external stress fields. The influence range of the abutment pressure of the super high mining height working face was measured on site. The numerical simulation is carried out, the roadway support structure is analyzed, and the improvement measures are proposed. The research results demonstrate that: The influence range of abutment pressure is 234 m, the obvious influence range of the leading pressure is 47–60 m, and the peak position of the influence of the leading pressure is 15–20 m. The 5 m range is the lateral inward stress field of the coal pillar, the 10–15 m range is the outward stress field of the coal pillar, and the 20 m range is the original rock stress field of the coal pillar. Therefore, it is proposed that the reasonable size of the coal pillar for roadway protection is 20–22 m. Adjusting the distance between screw steel and FRP bolts from 1000 mm to 1200 mm, the length of the roof prestressed anchor cable should be appropriately reduced to 5.5–6 m according to the lithology of the roof.

## 1. Introduction

The mining of large mining height coal seams mainly presents the following characteristics: the height of the machine mining increases, the control of the roof is more difficult, and the roof pressure occurs. There are frequent accidents of the frame, the surrounding rock is greatly affected by mining, and the surrounding rock is seriously deformed. Special geological engineering problems caused by special mining methods have had a serious impact on production. After mining, the roof activity law is not fully grasped, resulting in poor supporting effects of the adopted supporting methods. There is an urgent need to carry out relevant research to reveal the mining conditions of large mining heights. Based on the characteristics of special engineering geology, targeted support methods are proposed.

In terms of stope pressure theory, many experts have put forward new theories and hypotheses on the overburden pressure distribution and strata control of the stope. The propose theoretical computational model of abutment pressure for ETAS longwall panels is based on the analysis of load transfer mechanisms of key stratum (KS) and ETAS [1]. The established three-dimensional dynamic prediction model of the abutment pressure is based on elastic foundation theory [2]. The development and formation of ground fissures at panel 12,401 of the Shangwan Colliery in the Shendong mining area was studied and investigated, and the development and formation mechanisms of ground fissures through theoretical analysis and physical simulation were elucidated [3]. Relevant research on the horizontal stress distribution and stress variation range after mining was conducted [4,5]. The characteristics of multi-coal strata movement caused by downward longwall coal mining activities were studied [6]. The evolution characteristics of the composite pressure-arch in thin bedrock of overlying strata during shallow coal mining was researched [7]. A model is made for the Bayindir lead-zinc mine, which is working with room and pillar method and is about to be completed [8]. The typical strong rock pressure mine in China—an extra-thick alluvial mine, was studied [9,10]. The evolution of the peak abutment pressure in the shallow-buried large mining height face was studied [11]. The loose layer arch structure and its impact on the mining overburden was studied [12]. The deformation characteristics of the surrounding rock of roadway in the inclined coal seam goaf side and half coal rock roadway under four basic roof fracture forms are studied by using the numerical simulation method [13]. A self-designed model of the isolated pillar with an equivalent mining height and a monitoring system of stress is employed to study the progressive failure of the overlying strata and the changing rules of the induced displacement and stress [14]. An in-depth research on roadway support theory, support materials, and structure was conducted [15,16]. Extensive true-triaxial unloading tests are conducted on rectangular Miluo granite specimens to investigate their crack propagation behavior and peak unloading strength properties [17]. The incremental iterative calculation format of the Mogi-Coulomb strain-softening failure criterion was implemented in the program [18]. Reasonable layouts of large-section chambers were determined by analyzing the control effect of the stress shell on the surrounding rock under three kinds of in situ stress fields [19]. The failure characteristics of a deep circular tunnel in a rock mass with multiple weakness planes using a 2D-combined finite element method/discrete element method (FEM/DEM) were investigated [20]. The particle breakage of tailings was analyzed qualitatively and quantitatively [21]. A novel approach was proposed to quantify the separation and fracture evolution in the undermined overburden [22]. The Hilbert-Huang transform (HHT) method is used to obtain detailed structural characteristics of coal rock masses associated with damage, at different loading stages, from the analyses of the characteristics of AE waveforms, and researched the relationship between energy accumulation and dissipation during the coal rock dynamic deformation and rupture process to obtain the rockburst energy condition [23,24]. The mechanism of roof step-form subsidence caused by longwall mining in the Shendong shallow coalfield was determined [25]. In-depth studies on key layers of overburden in shallow coal seams were conducted. A lot of research on discrimination and control has been carried out [26,27]. Based on the distinguishing characteristics in the fully mechanized solid backfill mining technology, the basic principles and methods for mining pressure monitoring were analyzed and established [28]. Physical simulation to monitor and analyze the internal pressure of goaf was used, and it was found that goaf pressure presented a stepwise growth with the key stratum breaking [29]. The deep mines of Linyi Mining Area were considered as the research object, the stress distribution law of each mine was studied, and a model of elastic rectangular cantilever thin plates was established; the critical conditions for bending and fracturing the regenerated roof during mining were analysed [30,31]. A combined support methodology aiming at increasing the stability and safety of soft-rock roadways in the Zhongguan iron mine in China via an analysis of the field structure of weak surrounding rock by means of numerical modelling was investigated [32]. The caving zone presents obviously dynamic distribution characteristics in goaf with a large mining height. The changing height of the caving zone is related to the thickness and bulk density of the rock strata structure, mining time of workface, initial hulking coefficient and compression modulus of caving gangue [33]. Cyclic weighting in the super high mining face has the characteristics of short weighting steps, long duration of pressure, obvious regional weighting and strong dynamic rock pressure, and so on [34]. Uniaxial compression tests were performed on rocks with double holes and fractures at different angles, the failure behavior and mechanical properties of rock samples with holes and fractures at different angles were analyzed [35]. The above-mentioned studies have studied the distribution of the supporting stress in the overburden during coal mining by proposing or improving the existing rock pressure theory, and have accumulated a wealth of theoretical results. However, there are relatively few related studies on super large mining heights, so there is still a need for theoretical exploration, and improvements are being carried out on the distribution law of abutment pressure in the super large mining height working face.

In terms of mine pressure monitoring and control, the commonly used methods include field measurement and indoor physics, as well as numerical simulation. This innovated the theory and method of mine pressure monitoring [36]. The stability of the pillar in the stope was studied [37]. The FLAC3D numerical simulation analyzes the influence of mining height and depth on the abutment pressure of the large mining height working face [38]. The structure of the “short cantilever-articulated rock beam” and calculation formula of the support working resistance was resulted in [39]. A case study of the surrounding rock deformation characteristics and control technology of a typical gob-side coal-rock roadway in inclined coal seams in Guizhou, China [40]. An accurate laying of model and precise excavation of roadway method, named “labeling positioning and drawing line, presetting roadway model” was proposed; the physical similarity simulation of deformation and failure characteristics of the surrounding rock of coal-rock rise, under the influence of repeated mining in close distance coal seams, was carried out [41]. The development of fractures in the overburden strata above the goaf was simulated and the diffusion behaviors of the grout slurry under different conditions was studied [42]. The current situation was summarized and the characteristics of the underground HIM using the analytic hierarchy process (AHP) method in thick coal seams in China was analyzed [43]. Structural and movement characteristics of the overlying strata are investigated in different stages to reveal the strata subsidence control mechanism of backfill-strip mining [44]. The particle flow discrete element software PFC2D and laboratory experiments were used to conduct uniaxial compression tests on rocks with cracks of different angles around the hole [45]. It was found that there are two structural forms and six movement forms in the key layers of a fully mechanized stope with large mining height through research [46]. The research method in this aspect is mainly based on the force and deformation of the supporting structure measured on site, and the super mining is based on the abutment pressure theory. There are few studies on the numerical simulation of high supporting structures.

Experts at home and abroad have realized that the roof breakage of shallow mining directly affects the surface. The roof break angle is large, the surface sinks quickly, and the pressure is obvious and difficult to control. The mechanics analysis of this kind of rock formation movement theory and the direct simulation research through numerical simulation software are the lack of research on the abutment pressure field system of the super large mining height face. The relevant support technology is also limited by the slow development of theory and limited to traditional conventional support methods. It only increases the density without fundamental changes. At present, a lot of exploration and practice have been carried out in the research fields related to the working face abutment pressure and rock movement in China, but the research on large mining height working face is obviously insufficient, and there is little research on the spatial evolution of overlying strata and stress field characteristics of the large mining height mining site, as well as a lack of systematic and scientific theoretical guidance.

Based on the engineering background of the large mining height mining in the upper 108 working face of Jinjitan Coal Mine 12-2, through theoretical prediction, numerical simulation and engineering measurement and other research methods, the spatial structure of the super large mining height is determined, and the abutment pressure distribution law in the super large mining height mining is determined. The characteristics and surrounding rock control mechanism are studied, and the deformation of the roadway and supporting structure is analyzed, and corresponding supporting suggestions are put forward. The high-end surface leakage and step sinking of the shallow-buried super large mining height is effectively controlled, and the occurrence of related accidents in the super large mining height mining is prevented. The research of this project will provide technical support for in the safe mining of a fully mechanized mining face with large mining height (8.2 m support), and fill the gap of the research results of safe and efficient mining in the Yushen coal field and even the whole country. It is of great significance to improve the recovery rate of coal resources in the Yushen mining area and to achieve high yield and high efficiency, and also has a guiding significance to achieve high yield and high efficiency in the working face under similar conditions in China.

## 2. Theoretical Analysis of the Abutment Pressure Distribution Law of Super Large Mining Height Face

### 2.1. Spatial Structure of Abutment Pressure Development and Change

Chinese scholars have conducted a lot of work in the research of the mine pressure of the longwall working face. Academician Qian Minggao and Academician Song Zhenqi established the masonry beam theory [47] and the transmission rock beam theory, respectively [48]. The masonry beam theory focuses on the structural form of the rock beam, while the transfer rock beam theory focuses on the movement and failure process of the rock beam. The transfer rock beam theory also emphasizes the structural model and coordination between the advancing direction of the working face and the longitudinal rock beam. In terms of the theory of the large mining height ground pressure, China mainly use academician Qian Minggao’s “masonry beam” structure theory and academician Song Zhenqi’s “transmission rock beam” practical ground pressure theory system.

**Figure 1:**
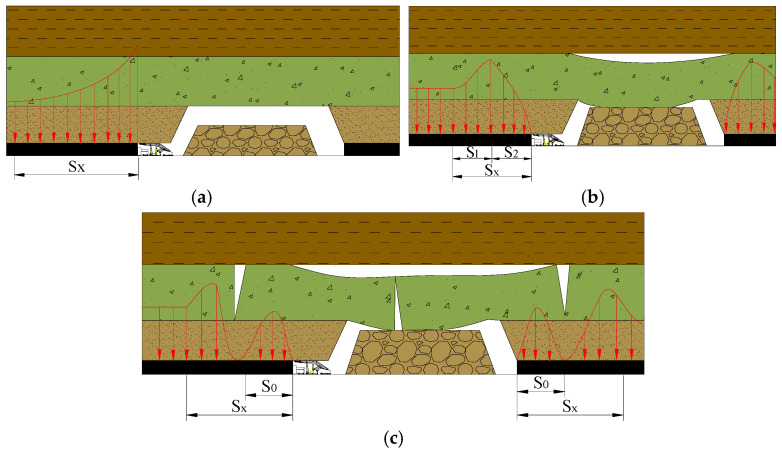
Development stage of abutment pressure. (**a**) Maintains elastic supporting capacity; (**b**) Loses elastic supporting capacity; (**c**) Formation of internal and external stress fields.

Theoretical research and field practice have proved that from the start of stope advancement to the end of the first controlled rock formation (fall zone and basic roof), the abutment pressure and its apparent changes can be divided into three stages (Figure 1).

#### 2.1.1. The First Stage of the Development and Change of Abutment Pressure—Coal Wall Maintains Elastic Supporting Capacity

Starting from the opening of the cut, the overhanging area of the roof has also been increasing, and the pressure transmitted by the coal walls on both sides of the stope through the roof rock has also gradually increased. Because the coal walls have a certain degree of hardness and strength, the stope has a certain degree of hardness and strength. Before the pressure transmitted by the roof reaches the limit of coal destruction, the entire coal wall is in an elastic compression state, and the abutment pressure distribution is a monotonous decreasing curve with a peak at the coal wall (Figure 1a); *S_x_* means the distribution range of abutment pressure. At this stage, the abutment pressure distribution range *S_x_* is relatively small. The coal wall maintains its inherent supporting capacity, and the coal wall in front of the stope is always in a state of elastic deformation, and the coal wall is not prone to roof leakage and fragmentation.

#### 2.1.2. The Second Stage of the Development and Change of Abutment Pressure—Coal Wall Loses Elastic Supporting Capacity

As the working face continues to advance, the exposed area of the roof further increases, and the pressure transmitted to the coal wall through the roof also gradually increases. With the increase in the tangential stress of the coal wall, the coal wall reaches its elastic support limit, and plastic deformation and even failure deformation begin to occur. The peak of the abutment pressure will gradually shift to the inner side of the coal wall until a new stress balance is reached (Figure 1b); *S_x_* means the distribution range of abutment pressure; *S_1_* means the plastic zone. *S_2_* means the elastic zone. This stage starts from the change in the supporting capacity of the coal wall and ends before the end of the rock beam at the lower level of the fracture zone breaks. The distribution of the abutment pressure on the coal seam will be divided into two intervals: the pressure in the plastic zone (the coal body has been completely destroyed) gradually rises, the pressure in the elastic zone drops monotonously, and the junction of the elastoplastic zone is the pressure peak position. The abutment pressure distribution range also consists of two parts, the plastic zone and the elastic zone.

#### 2.1.3. The Third Stage of the Development and Change of Abutment Pressure—Formation of Internal and External Stress Fields

From the end of the lower rock beam in the fractured group to the middle of the rock beam, it hits the gangue. Before the end of the rock beam fractured, the pressure was highly concentrated near the fracture line; after the end of the rock beam fractured, the abutment pressure distribution was clearly divided into two parts with the fracture line as the boundary, that is, between the fracture line and the coal wall by the arch. The “internal stress field” (σ < γH) was determined by the weight of the broken rock beam, and the “external stress field” (σ > Hγ) was determined by the overall weight of the overlying rock outside the fracture line (Figure 1c); *S_x_* means the distribution range of abutment pressure; *S_0_* means the range of internal stress field.

**Figure 2:**
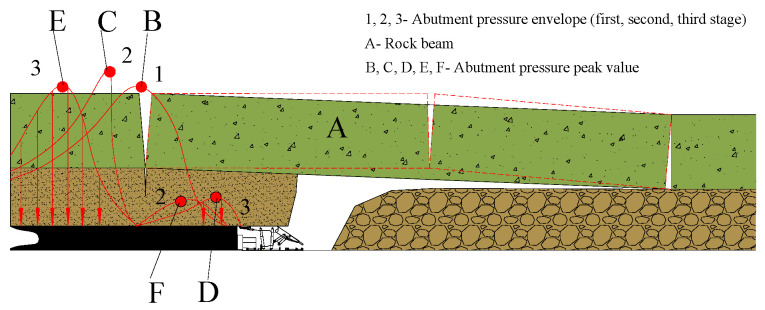
The forming process of internal and external stress field.

Before the rock beam A is fractured, the abutment pressure distribution curve is in front of the working face (Figure 2). The peak of the abutment pressure is concentrated near the fracture line B, and the force point of the rock beam A is also at the B point. When the rock beam A breaks, the abutment pressure changes rapidly. With point B as the dividing line, the abutment pressure divides into two peaks and shifts in opposite directions. The front shifts to point C, and the rear shifts to point D. As mentioned, point C is the boundary point of the elastoplastic zone outside the abutment pressure (i.e., the external stress field), and point D is the pressure peak on one side of the abutment pressure (i.e., the internal stress field).

With the rock beam A from the initial fracture to the stop of movement, the abutment pressure distribution also slowly changes from curve 2 to the state of curve 3, and the peak value will also go through the change process of B→C→E. At this time, similar changes will occur in the coal wall before the work and the coal wall behind the open cut.

### 2.2. Distribution Range of Coal Wall Abutment Pressure around the Working Face

#### 2.2.1. Bearing Pressure Distribution Range

According to the related literature [11,31], the distribution range of stope abutment pressure can be equivalent to the following equation:

(1) $$\int_{0}^{S_{x}}\sigma_{x}dx=\frac{S_{1}}{2}k\gamma_{p}H+\frac{\left(\gamma_{p}H+k\gamma_{p}H\right)}{2}S_{2}$$

where *S*_1_ is the range of the plastic zone; *S*_2_ is the range of the elastic zone; *Sx* is the distribution range of the abutment pressure on the coal body; *σ_x_* is the abutment pressure; *k* is the stress concentration factor; *γ_p_* is the bulk density of the stope overlying rock; *H* is the mining depth.

Generally with *k* = 2.5 and *L_x_* = *L*_0_, there are

(2) $$V_{cx}=\frac{1}{2}H\gamma_{p}\left[S_{b}+\left(L_{0}+2S_{x}\right)\right]-\frac{\left(L_{0}-2C_{i}\right)\gamma_{p}L_{0}}{2\times 2}$$

where *Vcx* is the volume of the cube; *L*_0_ is the length of the working face; *Sx* is the distribution range of the abutment pressure on the coal bodies on both sides of the working face.

The balance equation shown in Equation (2) is:

(3) $$2\int_{0}^{S_{x}}\sigma_{x}dx=\frac{1}{2}H\gamma_{p}\left[S_{b}+\left(L_{0}+2S_{x}\right)\right]-\frac{\left(L_{0}-2C_{i}\right)\gamma_{p}L_{0}}{2\times 2}=2\times \frac{13}{8}\gamma_{p}HS_{x}$$

(4) $$S_{b}=2S_{x}+L_{0}+2B$$

(5) B=Hcotθ

Simultaneous Equations (3)–(5) give the abutment pressure distribution in Equation (6)

(6) $$S_{x}=\frac{4\left(L_{0}+H\cos\theta \right)}{5}-\frac{L_{0}\left(L_{0}-2C_{i}\right)}{5H}$$

#### 2.2.2. Internal and External Stress Field Range

After the end of the rock beam at the lower position of the fracture group is fractured, the abutment pressure distribution is divided into two parts: the “internal stress field” between the fracture line and the coal wall determined by the weight of the fractured rock beam in the arch, and the “external stress field” outside the fracture line is determined by the overall weight of the overlying strata. The calculation of the internal stress field is based on the fact that the vertical abutment pressure distributed in the internal stress field on the coal body around the goaf is equal to the weight of the basic roof beam (slab) before the initial pressure on the working face.

(7) $$\frac{1}{2}S_{0}\frac{K_{\max}\gamma HS_{0}}{S_{1}}=\frac{H_{g}\cdot C_{i}\cdot \gamma }{2}$$

where *S*_0_ is the internal stress field range; *C_i_* is the basic top rock beam periodic compression step; *K_max_* is the stress concentration factor; *H* is the mining depth; *S*_1_ is the distance between the peak position of the abutment pressure and the coal wall; *γ* is the rock mass density.

The calculation Equation (8) for the range of the internal stress field is obtained by solving Equation (7)

(8) $$S_{0}=\sqrt{\frac{C_{i}\cdot H_{g}\cdot S_{1}}{K_{\max}H}}$$

## 3. The Actual Measurement of the Mine Pressure on the Super Large Mining Height Face

### 3.1. Project Overview

**Figure 3:**
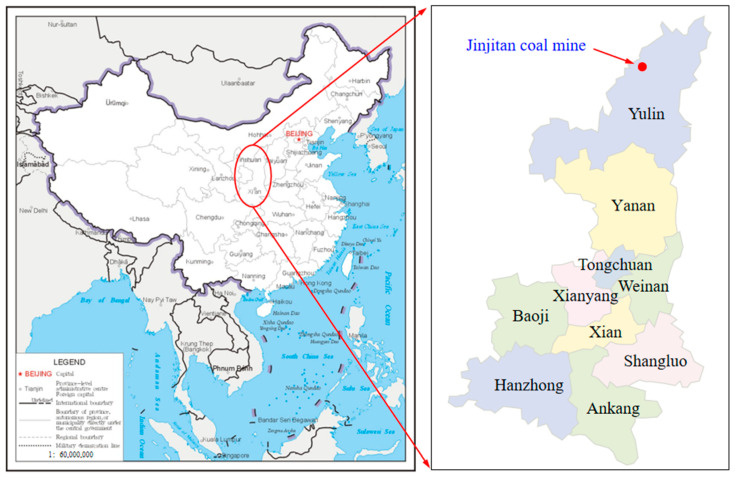
Location map of Jinjitan coal mine.

The Jinjitan coal mine field is located on the northeastern edge of the Ordos Basin, a Jurassic coalfield in northern Shaanxi, in the south of the Yushenfu coalfield in northern Shaanxi. It is located in the transition zone between the edge of the Mu Us Desert and the Loess Plateau. The surface is covered by thick loose aeolian sand. The thickness of the 2-2 upper coal seam is 5.5–8.4 m, with an average of 6.65 m, the buried depth of the coal seam is 200–305 m, the average mining depth is 246 m, and the average thickness of the loose layer is 35 m, which belongs to the near shallow coal seam. It is suitable for the arrangement of the super large mining height working face. The location of Jinjitan Coal Mine is shown in Figure 3.

2-2 Upper coal seam 108 working face is the first mining working face in the southwest wing of a panel, with an inclination length of 300 m, a strike length of 5527.4 m, and an average inclination angle of less than 1°. Occurrence of coal seams: The thickness of the coal seams is 5.5–8.4 m, with an average of 6.65 m. The overall structure is a monoclinic structure, high in the northeast and low in the southwest; the average inclination of the coal seam is 0.5°.

The cut hole of the 108 working face is tunneled along the 2-2 coal seam floor. The sandstone fissure water of the Zhiluo and Yan’an formations within the caving fissure zone was formed by mining, and the old goaf water formed after mining will be the mining process of the working face of the main water hazard. The cumulative thickness of aeolian sand and Sarawusu Formation within the working face is 9.50–29.40 m, and the thickness of red clay is 0–22.65 m, which is missing in the cut-off section, middle part and near the stop line of the working face; the thickness of the loess is 0–20.89 m; the thickness of Luo Formation sandstone is 106.20–152.45 m, of which the weathering zone is continuously distributed on the top of the bedrock, and the thickness is 21.93–54.70 m; the coal seam is overlying the fifth member of Yanan Formation, the thickness is 25.10–57.86 m, and the average is 47.20 m.

### 3.2. Theoretical Calculation of Abutment Pressure Distribution Range

**Figure 4:**
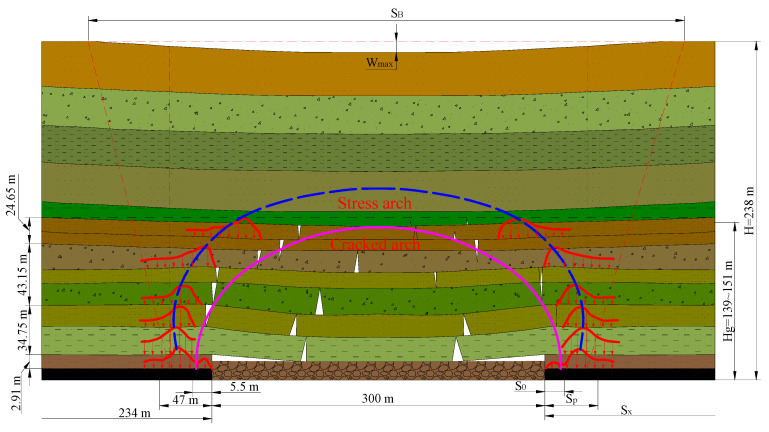
Main parameters of surrounding rock structure mechanics of 108 working face.

According to the relevant theoretical research on mine pressure and rock formation control, combined with the specific parameters of the 108 working face on 12-2 of the Jinjitan Coal Mine, the slope length is 300 m, the initial pressure step is 98–117 m, and the periodic pressure step is 22 m on average. The depth is about 258 m, and the rock formation movement angle is about 75°. Substituting the data into Equations (6) and (8), it can be obtained that the bearing pressure distribution range is about 234 m, the apparent range of bearing pressure is about 47 m, and the range of the internal stress field is about 234 m. It is 5.5 m, the impact range of the peak abutmentt pressure is 15–20 m, and the peak point of the maximum abutment pressure is about 6–10 m in front of the coal wall. According to the theoretical research and combined with the abutment pressure distribution range, the surface subsidence range of 108 working face can be calculated as 1917.86 mm (Figure 4). The theoretical calculation of the bearing pressure is shown in Table 1.

It can be observed from Table 1 that the abutment pressure and the leading pressure are 234 m and 47 m, respectively, the range of the internal stress field is within 5.5 m, and the leading pressure peak position is 15–20 m. It can be observed that the abutment pressure and the leading pressure range of the super large mining height working face are larger than the ordinary pressure range.

### 3.3. Measuring Point Layout

**Figure 5:**
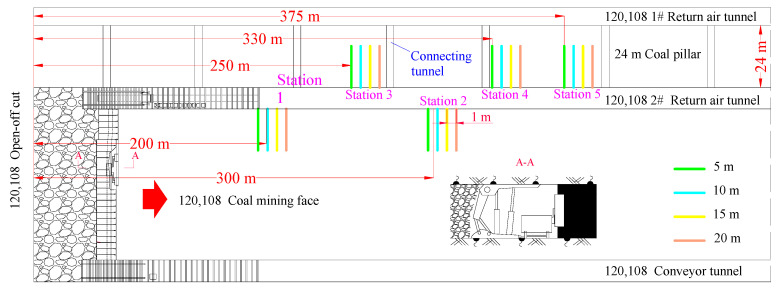
Installation diagram of flexible detection unit.

The 108 working face adopts the fully mechanized mining method, and the working face is equipped with hydraulic support, shearer and scraper conveyor. The on-site observation layout of the abutment pressure is a flexible detection unit for a fully mechanized mining face with 108 mining heights (Figure 5). The first and second measurement stations are located on the side of the coal body, and the third, fourth, and fifth measurement stations are located on the side of the coal pillar.

(1) The abutment pressure monitor for the roadway on the side of the 108 working face is equipped with five measuring stations. The first measuring station is arranged on the side of the solid coal 200 m and 300 m away from the open cut. The depths are 5 m, 10 m, 15 m, and 20 m, and the spacing is 1 m.
(2) The third station, the fourth station, and the fifth station are arranged at 250 m, 330 m, and 375 m away from the open cut hole. Each station has 4 measuring points, and the hole depths are 5 m, 10 m, 15 m, 20 m, spacing 1 m.
(3) Holes are drilled horizontally, and the hole diameter is between 46 mm.
(4) The hole height is 1.5 m from the bottom plate.

### 3.4. Test Results

**Figure 6:**
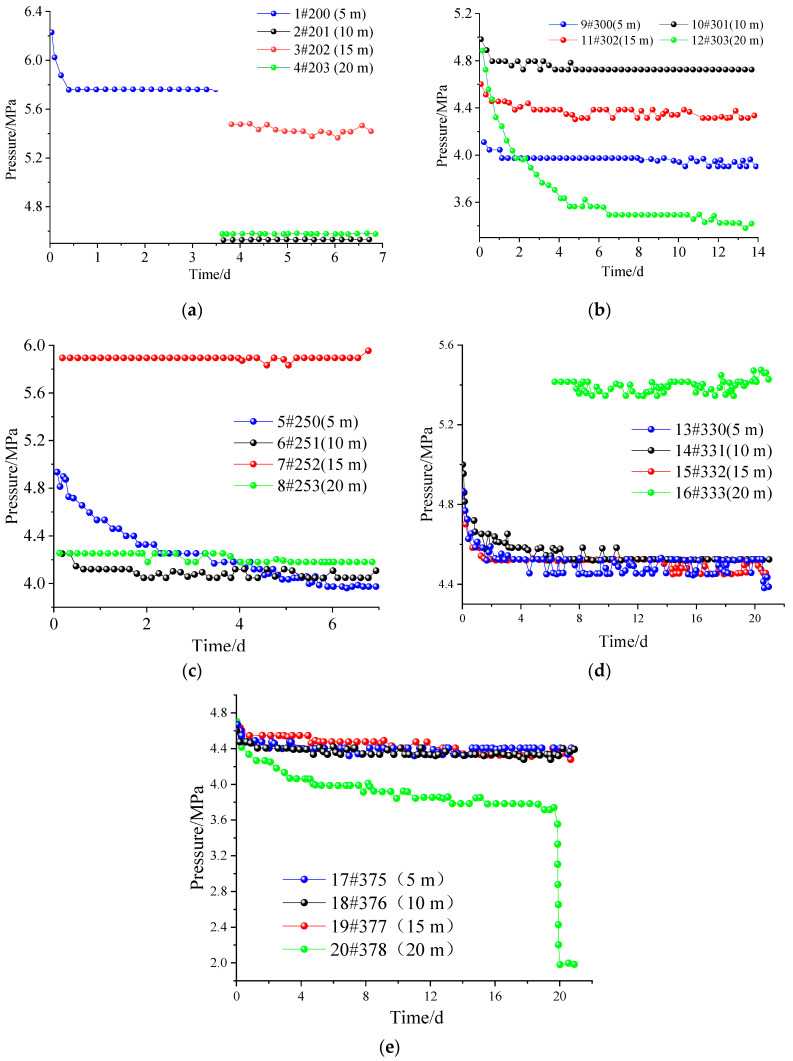
Stress monitoring diagram. (**a**) The first station; (**b**) The second station; (**c**) The third station; (**d**) The fourth station; (**e**) The fifth station.

According to the monitoring data from the first to fifth stations in Figure 6, the pressure changes at the 10 m and 15 m measurement points are small and there is no continuous pressure drop. Therefore, it can be determined that the 10–15 m range should be in the outer stress field from the side of the coal body, as a 5 m measurement. The point has been in a depressurization state, so it can be determined that the 5 m range should be in the lateral internal stress field range of the coal body. According to the third, fourth, and fifth measuring stations, the pressure changes at 10 m and 15 m measuring points are small, so it can be determined that the range of 10–15 m should be in the range of the outward stress field on the coal pillar side; the 5 m measuring point has been in a depressurization state, so it is determined that the 5 m range should be in the lateral internal stress field range of the coal pillar; the 20 m measurement point has been in the depressurization state, so it can be determined that the 20 m range should be within the original coal pillar stress field. Therefore, the measured ground pressure is basically the same as the theoretical analysis. The correctness of the theory can be proved by comparing the measured data with the theoretical calculated value.

According to the monitoring data from the first to fifth stations, the abutment pressure near the coal mining face is significantly increased. For the coal body of the stope, the internal and external stress fields can be divided. For the coal pillar, the abutment pressure near the roadway decreases. As the working face advances, the peak abutment pressure shifts to the depths of the coal pillar.

## 4. Numerical Simulation of Supporting Structure of Super Large Mining Height Working Face

### 4.1. Numerical Model Establishment

The finite element FLAC3D is used for simulation. The numerical model simulates the mechanical parameters of each layer of rock mass, based on the columnar diagram of the 108 working face, the geological section diagram, and the rock mechanical parameters. Horizontal constraints are imposed on the front and rear, left and right boundaries of the model and horizontal and vertical constraints are imposed on the bottom boundary of the model, and the equivalent load in the vertical direction is imposed on the upper boundary of the model (*P = γH*), which represents the weight of the overlying strata of the model, where *γ* is the overlying model. The average volume force of the rock formation is taken as 0.023 MN/m^3^; *H* is the distance from the upper part of the model to the surface.

**Figure 7:**
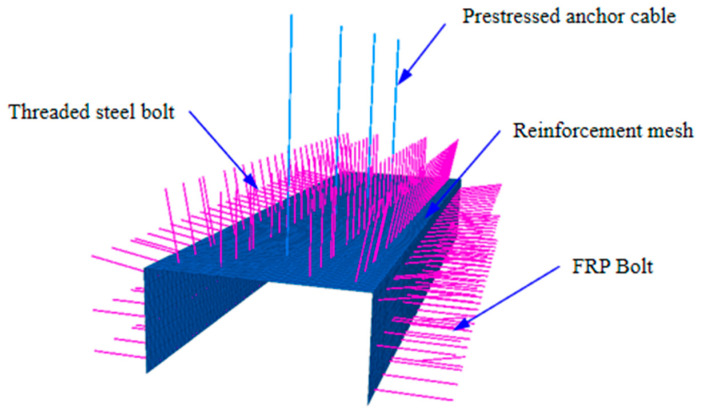
Realization method of 3D modeling support structure simulation.

In the numerical calculation, the Cable element is used to simulate the prestressed anchor cable, the Beam element is used to simulate the threaded steel anchor rod and the glass fiber-reinforced plastic anchor rod, and the Shell element is set to simulate the steel mesh. The key to the simulation of the prestressed anchor cable lies in the application of prestress (Figure 7). The FLAC3D simulation of coal seam excavation in the mining area is implemented using a null model. In order to complete the excavation, only the material model of the excavation grid body needs to be set to a null model.

### 4.2. Simulation Results

#### 4.2.1. Roadway Deformation Analysis

**Figure 8:**
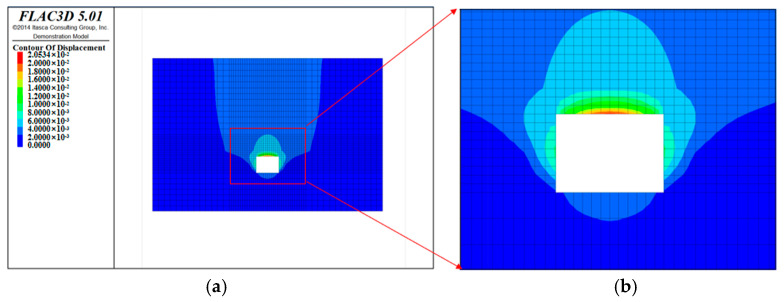
Cloud chart of surrounding rock deformation. (**a**) Overall picture; (**b**) Enlarged picture.

**Figure 9:**
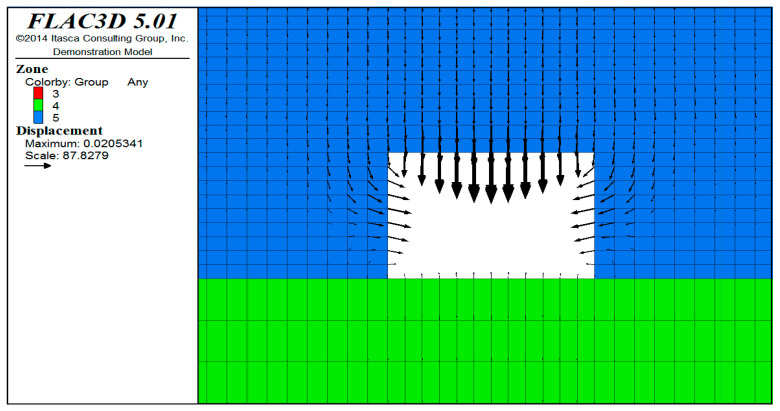
Vector diagram of deformation and displacement.

From the surrounding rock deformation cloud map (Figure 8) and the deformation displacement vector diagram (Figure 9), it can be observed that with the advancement of the working face, the deformation of the surrounding rock of the roadway gradually increases; in particular, the deformation value near the vault is the largest, and the maximum value is 20.5 mm. The vault extends upwards, and the stratum deformation converges quickly. After extending 5 m into the rock body, the surrounding rock deformation is basically within 5 mm.

**Figure 10:**
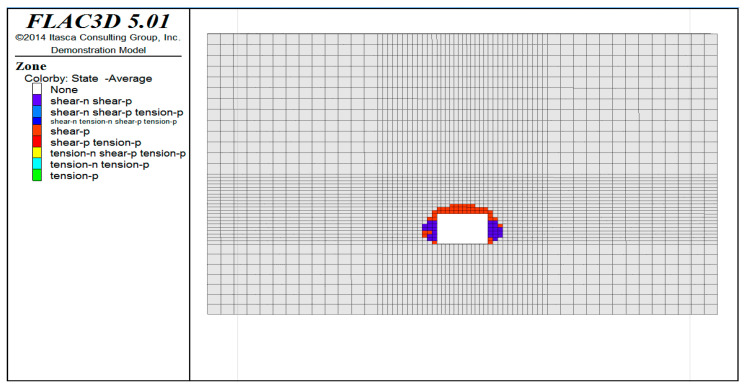
Distribution of plastic zone.

From the distribution map of the plastic zone (Figure 10), it can be observed that the maximum depth of the plastic zone is about 1.8 m, which is mainly located in the side wall and vault of the roadway. There is no plastic zone near the bottom plate. The side wall is mainly a shear plastic zone, while the vault is the shear–stretch plastic zone. In general, the development depth of the plastic zone is not large, the distribution range is also small, and the surrounding rock stability of the roadway construction is good.

#### 4.2.2. Force Analysis of Supporting Structure

**Figure 11:**
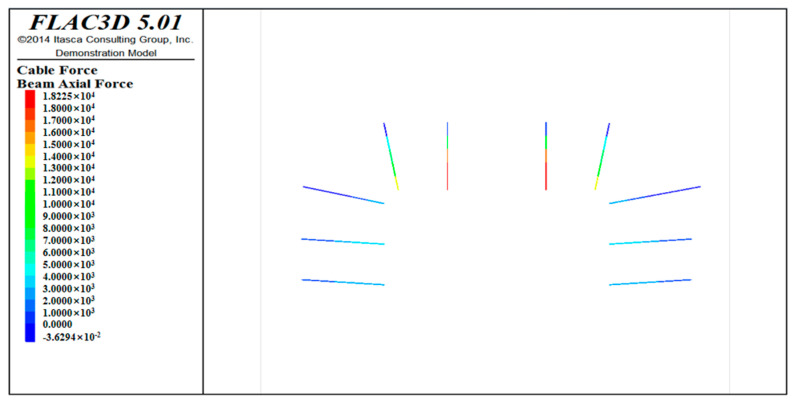
Stress distribution of anchor rod.

From the anchor force distribution diagram (Figure 11), it can be observed that the tensile force of the anchor rod at the dome is the largest, with a maximum value of 28.4 kN (tensile stress value of 111.66 MPa), and the anchor rod far away from the dome is relatively small, as it is mainly between 10–20 kN. The force of the bolt is mainly derived from the deformation of the surrounding rock. The deformation law of the surrounding rock shows that the position of the bolt can be adjusted appropriately, as close as possible to the vault. In addition, the force of the bolt is relatively small and can be reduced the bolt spacing. The prestress of the prestressed anchor cable is 100 kN. With the excavation of the roadway, the force of the prestressed anchor cable gradually increases. The maximum force is 140.85 kN, which can meet the force requirements (the prestressed anchor cable can withstand the maximum tensile force of 200 kN).

#### 4.2.3. Supporting Design Suggestions

(1) With the advancement of the working face, the deformation value near the vault is the largest, and the maximum value is 20.5 mm. The farther away from the center of the vault, the smaller the deformation of the surrounding rock. The bolt force is closely related to the deformation of the surrounding rock. The anchor rod is relatively small; thus, it is recommended that the anchor rod of the top arch should be appropriately close to the center of the dome.
(2) The deformation of the surrounding rock is small, and the plastic zone of the surrounding rock is not deep; thus, the bolts are not stressed. Therefore, you can consider adjusting the distance between the rebar and FRP bolts from 1000 mm to 1200 mm.
(3) Since the deformed vault of the surrounding rock is relatively large and the two sides are relatively small, the two sides are relatively stable. Therefore, it can be considered to appropriately reduce the height of the hanging nets at the two sides of 1 m.
(4) The depth of the plastic zone is 1.8 m, and the larger deformation of the rock mass mainly occurs in the plastic zone. The force of the prestressed anchor cable is only 140.85 KN, which can appropriately reduce the length of the prestressed anchor cable to 5.5–6 m.

## 5. Conclusions

Aiming at the problem of the reasonable abutment pressure in shallow-buried, large mining height, and thick coal seams, this paper adopts theoretical analysis, field measurement, and numerical simulation methods, taking Jinjitan Coal Mine as an example to conduct research, and the main conclusions are as follows:

(1) During the mining process of a shallow coal seam with a large mining height face, the abutment pressure and its apparent changes can be divided into: the coal wall maintains its elastic supporting capacity, the coal wall loses its elastic supporting capacity, and the internal and external stress fields are formed.
(2) Use elastic mechanics, mine pressure, and rock formation control theory to establish a three-dimensional structural mechanics’ model of stope abutment pressure, and calculate the internal and external stress field range of the abutment pressure according to the model. The range of abutment pressure (*S_x_*) is 234 m, the range of leading pressure (*S_p_*) is 47 m, the range of internal stress field (*S_0_*) is 5.5 m, and the range of the leading pressure peak position is 15–20 m.
(3) According to the data of the coal body measurement points on both sides of the working face along the channel, the results demonstrate that the bearing pressure influence range is 234 m, the internal stress field range is 5.5 m, and the 10–15 m range should be in the coal pillar side outward stress field range. The obvious influence range is 47–60 m, and the peak position of the leading pressure influence is 15–20 m.
(4) Establish a numerical model of roadway deformation and support structure forces, and optimize the design of roadway support parameters according to the simulation results. You can consider adjusting the spacing between rebar and FRP bolts from 1000 mm to 1200 mm; the roof prestressed the anchor cables. The length can be appropriately reduced to 5.5–6 m according to the roof lithology.

## Figures and Tables

**Table 1:** Theoretical prediction of abutment pressure distribution in 108 working face.

Project	Range/m
Abutment pressure (*S_x_*)	234
Leading pressure (*S_p_*)	47
Internal stress field (*S_0_*)	5.5
Leading pressure peak position	15–20

## Data Availability

All data of this study have been included in the text.

## References

[1] Zhu S.T., Feng Y., Jiang F.X. (2016). Determination of Abutment Pressure in Coal Mines with Extremely Thick Alluvium Stratum, A Typical Kind of Rockburst Mines in China. Rock Mech. Rock Eng..

[2] Zhang Z.M., Kang T.H. (2021). Prediction Model of Abutment Pressure Affected by Far-Field Hard Stratum Based on Elastic Foundation Theory. CMC-Comput. Mater. Con..

[3] He X., Zhao Y.X., Yang K., Zhang C., Han P.H. (2021). Development and formation of ground fissures induced by an ultra large mining height longwall panel in Shendong mining area. B. Eng. Geol. Environ..

[4] Majdi A., Hassani F.P., Nasiri M.Y. (2012). Prediction of the height of destressed zone above the mined panel roof in longwall coal mining. Int. J. Coal Geol..

[5] Suchowerska A.M., Carter J.P., Merifield R.S. (2014). Horizontal stress under supercritical longwall panels. Int. J. Rock Mech. Min. Sci..

[6] Cheng G.W., Yang T.H., Liu H.Y., Wei L.K., Zhao Y., Liu Y.L., Qian J.W. (2020). Characteristics of stratum movement induced by downward longwall mining activities in middle-distance multi-seam. Int. J. Rock Mech. Min. Sci..

[7] Alexander V., Davide E., Douglas S. (2010). Role of Rock Mass Fabric and Faulting in the Development of Block Caving Induced Surface Subsidence. Rock Mech. Rock Eng..

[8] Malli T., Yetkin M.E., Ozfirat M.K., Kahraman B. (2017). Numerical analysis of underground space and pillar design in metalliferous mine. J. Afr. Earth Sci..

[9] María B., Díaz A.C., González (2008). Influence of the stress state in a coal bump-prone deep coalbed: A case study. Int. J. Rock Mech. Min. Sci..

[10] Zhu X.J., Guo G.L., Liu H., Chen T., Yang X.Y. (2018). Experimental research on strata movement characteristics of backfill-strip ming using similar material modeling. B Eng. Geol. Environ..

[11] Huang Q.X., Du J.W., Chen J., He Y.P. (2021). Coupling control on pillar stress concentration and surface cracks in shallow multi-seam mining. Int. J. Min. Sci. Technol..

[12] Ghabraie B., Ren G., Zhang X.Y., Smith J. (2015). Physical modelling of subsidence from sequential extraction of partially overlapping longwall panels and study of substrata movement characteristics. Int. J. Coal. Geol..

[13] Gao L., Liu P.Z., Zhang P.D., Wu G.Y., Kang X.T. (2022). Influence of fracture types of main roof on the stability of surrounding rock of the gob-side coal-rock roadway in inclined coal seams and its engineering application. Goal Geol. Explor..

[14] Chen Y., Li D., Jiang F.X., Zhang L.L., Zhu S.T. (2020). Use of the equivalent mining height method for understanding overlying strata movement and stress distribution in an isolated coal pillar. Shock Vib..

[15] Zhang N., Zhang N.C., Han C.L., Qian D.Y., Xue F. (2014). Borehole stress monitoring analysis on advanced abutment pressure induced by Longwall Mining. Arab. J. Geosci..

[16] Cao J.C., Zhang N., Wang S.Y., Wei Q. (2021). Investigation of mechanical properties for group anchors. Appl. Sci..

[17] Feng F., Chen S.J., Wang Y.J., Huang W.P., Han Z.Y. (2021). Cracking mechanism and strength criteria evaluation of granite affected by intermediate principal stresses subjected to unloading stress state. Int. J. Rock Mech. Min. Sci..

[18] Feng F., Li X.B., Rostami J., Peng D.X., Li D.Y., Du K. (2019). Numerical investigation of hard rock strength and fracturing under polyaxial compression based on Mogi-Coulomb failure criterion. Int. J. Geomech..

[19] Zhu C., Yuan Y., Wang W.M., Chen Z.S., Wang S.Z. (2021). Research on the “three shells” cooperative support technology of large-section chambers in deep mines. Int. J. Min. Sci. Technol..

[20] Chen S.J., Feng F., Wang Y.J., Li D.Y., Huang W.P., Zhao X.D., Jiang N. (2020). Tunnel failure in hard rock with multiple weak planes due to excavation unloading of in-situ stress. J. Cent. South Univ..

[21] Chen Q.L., Yang C.H., Zhang C., Ma C.K., Pan Z.K. (2019). Mechanical behavior and particle breakage of tailings under high confining pressure. Eng. Geol..

[22] Wang S.F., Li X.B., Wang S.Y. (2017). Separation and fracturing in overlying strata disturbed by longwall mining in a mineral deposit seam. Eng Geol..

[23] Li X.L., Chen S.J., Liu S.M., Li Z.H. (2021). AE waveform characteristics of rock mass under uniaxial loading based on Hilbert-Huang transform. J. Cent. South Univ..

[24] Li X.L., Chen S.J., Li Z.H., Wang E.Y. (2021). Rockburst mechanism in coal rock with structural surface and the microseismic (MS) and electromagnetic radiation (EMR) response. Eng. Fail Anal..

[25] Wang C.L., Zhang C.S., Zhao X.D., Liao L., Zhang S.L. (2018). Dynamic structural evolution of overlying strata during shallow coal seam longwall mining. Int. J. Rock Mech. Min..

[26] Xu J.L., Zhu W.B., Ju J.F. (2014). Supports crushing types in the longwall mining of shallow seams. J. China Coal Soc..

[27] Li Z., Xu J.L., Ju J.F., Zhu W.B., Xu J.M. (2018). The effects of the rotational speed of voussoir beam structures formed by key strata on the ground pressure of stopes. Int. J. Rock Mech. Min. Sci..

[28] Zhang Q., Zhang J.X., Kang T., Sun Q., Li W.K. (2015). Mining pressure monitoring and analysis in fully mechanized backfilling coal mining face-A case study in chai chen coal mine. J. Cent. South Univ..

[29] Xie J.L., Xu J.L. (2019). The corresponding relationship between the change of goaf pressure and the key stratum breaking. J. Geophys. Eng..

[30] Li X.L., Chen S.J., Wang S. (2021). Study on in situ stress distribution law of the deep mine taking Linyi Mining area as an example. Adv. Mater. Sci. Eng..

[31] Li X.L., Chen S.J., Zhang Q.M., Gao X. (2021). Feng, F. Research on theory, simulation and measurement of stress behavior under regenerated roof condition. Geomech. Eng..

[32] Yu K.P., Ren F.Y., Puscasu R., Lin P., Meng Q.G. (2020). Optimization of combined support in soft-rock roadway. Tunn. Undergr. Space Tech..

[33] Xiang P., Sun L.H., Ji H.G., Gao Y., Liu Y.J., Wu Y.F. (2017). Dynamic distribution characteristics and determination method of caving zone in large mining height working face. J. Min. Saf. Eng..

[34] Yang J.Z., Liu Q.J. (2020). Measurement and Mechanism Analysis of Ground Pressure Behavior Law of 0.8m Super High Mining Face. Coal Sci. Technol..

[35] Pan H.Y., Jiang N., Gao Z.Y., Liang X., Yin D.W. (2022). Simulation study on the mechanical properties and failure characteristics of rocks with double holes and fractures. Geomech. Eng..

[36] An B.F., Miao X.X., Zhang J.X., Ju F., Zhou N. (2016). Overlying strata movement of recovering standing pillars with solid backfilling by physical simulation. Int. J. Min. Sci. Technol..

[37] Esterhuizen G.S., Dolinar D.R., Ellenberger J.L. (2010). Pillar strength in underground stone mines in the United States. Int. J. Rock Mech. Min. Sci..

[38] Jin Z.P., Qin T., Zhang J.W. (2018). Analysis of abutment pressure distribution characteristics and influencing factors of deep mining height face. Coal Sci. Technol..

[39] Yan S.H., Yin X.W., Xu H.J., Xu G., Liu Q.M., Yu L. (2011). Roof structure of short cantilever-articulated rock beam and calculation of support resistance in full-mechanized face with large mining height. J. China Coal Soc..

[40] Liu P., Gao L., Zhang P., Wu G., Wang C., Ma Z., Kong D., Kang X., Han S. (2022). A Case Study on Surrounding Rock Deformation Control Technology of Gob-Side Coal-Rock Roadway in Inclined Coal Seam of a Mine in Guizhou, China. Processes.

[41] Liu P., Gao L., Zhang P., Wu G., Wang Y., Liu P., Kang X., Ma Z., Kong D., Han S. (2022). Physical Similarity Simulation of Deformation and Failure Characteristics of Coal-Rock Rise under the Influence of Repeated Mining in Close Distance Coal Seams. Energies.

[42] Shi H., Zhang Y.B., Tang L. (2021). Physical test of fracture development in the overburden strata above the goaf and diffusion process of permeable grout slurry. B Eng. Geol. Environ..

[43] Bai E.H., Guo W.B., Tan Y. (2019). Negative externalities of high-intensity mining and disaster prevention technology in China. B Eng. Geol. Environ..

[44] Zhu W.B., Qi X.R., Ju J.F., Xu J.M. (2019). Mechanisms behind strong strata behaviour in high longwall mining face-ends under shallow covers. J. Geophys. Eng..

[45] Yao D.H., Jiang N., Wang X.J., Jia X.D., Lv K. (2022). Mechanical behaviour and failure characteristics of rocks with composite defects of different angle fissures around hole. B Eng. Geol. Environ..

[46] Liang Y.P., Li B., Yuan Y., Zou Q.L., Jia L.X. (2017). Moving type of key strata and its influence on ground pressure in fully mechanized mining face with large mining height. J. China Coal Soc..

[47] Qian M.G., Miao X.X., He F.L. (1994). Analysis of key blocks of stope “masonry beam” structure. J. China Coal Soc..

[48] Song Z.Q. (1978). Movement of overlying strata in stope and selection of supports. Coal Sci. Technol..

